# Dr. Frank S. Bourns

**Published:** 1901-01

**Authors:** 


### DR. FRANK S. BOURNS.

THE first political party under the American régime is in process of formation. Its principles have been embodied in a platform which will shortly be made public. It is understood declarations of the platform give the fullest recognition to American sovereignty, and also favor a considerable degree of native autonomy concerning internal and local affairs.

Several of the most intelligent Filipino leaders, who have been instrumental in bringing the matter to a head, have been in conference with those interested, and this evening the platform will be outlined to the Philippine commission by Senor Buencamino, former premier in the so-called government of Aguinaldo; Colonel Aquiles, and Dr. Frank S. Bourns, an American, formerly chief surgeon, with the rank as major, and health officer of Manila.

Dr. Bourns was with Professor Dean C. Worcester prior to the American occupation, and has confidential relations with the Filipino leaders.

The commissioners are not likely to give public expression to their views regarding the formation of political parties, but the principles of the new organization, so far as made known, seem to be favored by intelligent Filipinos.—*Atlanta Constitution*.

As Dr. Bourns, referred to in the above paragraph, was once a resident of Atlanta, it may interest our readers to learn something of his history.

Frank S. Bourns was born in Michigan thirty-four years ago, and is sprung from Scotch and English stock. His father is a minister of the Methodist-Episcopal faith, and is now filling a charge in Detroit. Dr. Bourns received his classical education at the University of Michigan at Ann Arbor, and while yet a student he accompanied Prof. Steere on his notable expedition to the Philippines, which was organized for the purpose of investigating the ornithology of the archipelago. Returning after a year's absence with the expedition, he reentered the Michigan University and received his baccalaureate degree. His experience in the Philippines aroused a dormant love for natural history, and the enthusiasm with which he pursued the study of zoology and the accuracy and originality of his work won for him more than local notice. It was not long before he was selected, along with Dean C. Worcester, now Adjunct Professor of Zoology at Ann Arbor, and one of the members of the Philippine commission, by the Minnesota Scientific Society, to return to the scene of his labors in the East in the interest of this body. The immediate object was a more extended study of the native fauna of the Philippine islands and Borneo. Three years were devoted to this object, visiting the principal islands of the Philippine group, and included a six months' exploration of the island of Borneo, during which time Dr. Bourns, unattended except by native followers, penetrated into hitherto unexplored jungles of that mysterious land, the home of the man-monkey, and the scarcely more human Dyak head-hunters. Dr. Bourns's work in connection with the migration of birds, and the discovery and naming of new species as the result of his observations, gained for him recognition in scientific circles, and reference to it may be found in standard text-books of ornithology, and especially in the publications on this subject from the British Museum and the Smithsonian Institution.

Returning to the United States, he entered the medical department of the University of Michigan and received his medical diploma in 1896. Some time after graduation he was appointed Professor of Pathology in the Southern Medical College of Atlanta, where, for two years, he filled the chair with great credit to the institution and to himself. But his bold and adventurous spirit ill-comported with the dull routine of the college duties, and when the war with Spain broke out he promptly tendered his services to the government, firmly but modestly convinced that his unusual advantages and opportunities, which had acquainted him intimately with the Filipino character, and had given him a thorough knowledge of their country, would be of value to the government.

His representations were strongly fortified by influential friends in Atlanta and elsewhere, and resulted in his appointment as surgeon with the rank of major, attached to the staff of General Wesley Merritt.

His energy, belief in himself, and special qualifications soon made themselves felt. He succeeded, as president of the Manila Board of Health, in suppressing smallpox by establishing detention and isolation hospitals, and in instituting compulsory vaccination. He organized the work of the health board into effective activity, established a vaccine farm, and was largely instrumental in rectifying the unsanitary condition in which the army of occupation found the city of Manila. He became the trusted lieutenant of General Otis, and was generally regarded as possessing more influence over the friendly Filipinos than any other American in the army. Such was the esteem in which he was held by the upper class of native citizens of Manila that before his return to the States he was publicly banqueted, and received all manner of complimentary attention at their hands, and on his departure was accompanied to his steamer by representative Filipinos, with bands of music and other evidences of lavish Spanish courtesy.

Being obligated to return to Atlanta, he resigned from the army, having been warmly praised by General Otis and President McKinley for his patriotic and efficient services. He remained in Atlanta during the winter of 1899–1900, and in April of this year he again sailed for the Philippines with the intention of engaging in commercial pursuits.

The paragraphs in the *Constitution* of the 18th give evidence of his continued rise.

Inflexibly honest, deliberate yet bold, energetic yet cautious, cool in head, clear in judgment, Dr. Bourns is a type of the American to whom the future of our nation may be securely entrusted, with the assurance that in their hands the management of her affairs will be performed with absolute fidelity to her interests, and their policy will be not that of dishonesty, greed and rapacity, but moderation, justice and wisdom.

The friends of Dr. Bourns on this side of the Pacific will watch his career with an interest which time may not extinguish nor separation diminish, constantly stimulated by confidence and regard.

